# Comparison of the recovery rate of otomycosis using betadine and clotrimazole topical treatment^☆^

**DOI:** 10.1016/j.bjorl.2017.04.004

**Published:** 2017-05-06

**Authors:** Mohammad Reza Mofatteh, Zahra Naseripour Yazdi, Masoud Yousefi, Mohammad Hasan Namaei

**Affiliations:** a Birjand University of Medical Science, School of Medicine, Department of Ears, Nose and Throat, Birjand, Iran; b Birjand University of Medical Sciences, School of Medicine, Birjand, Iran; c Birjand University of Medical Science, Infectious Diseases Research Center, Birjand, Iran

**Keywords:** Otomycosis, Topical betadine, Topical clotrimazole, Recovery rate, Otomicose, Betadina tópica, Clotrimazol tópico, Taxa de recuperação

## Abstract

**Introduction:**

Otomycosis is a common diseases that can be associated with many complications including involvement of the inner ear and mortality in rare cases. Management of otomycosis can be challenging, and requires a close follow-up. Treatment options for otomycosis include local debridement, local and systemic antifungal agents and utilization of topical antiseptics.

**Objective:**

This study was designed to compare the recovery rate of otomycosis using two therapeutic methods; topical betadine (Povidone-iodine) and clotrimazole.

**Methods:**

In this single-blind clinical trial, 204 patients with otomycosis were selected using a non-probability convenient sampling method and were randomly assigned to two treatment groups of topical betadine and clotrimazole (102 patients in each group). Response to treatment was assessed at 4, 10 and 20 days after treatment. Data were analyzed using the independent *t*-test, Chi-Square and Fisher exact test in SPSS v.18 software, at a significance level of *p* < 0.05.

**Results:**

The results showed that out of 204 patients with otomycosis, fungi type isolated included *Aspergillus* in 151 cases (74%), and *Candida albicans* in 53 patients (26%). On the fourth day after treatment, 13 patients (13.1%) in the group treated with betadine and 10 patients (9.8%) in the group treated with clotrimazole showed a good clinical response to treatment (*p* = 0.75). A good response to treatment was reported for 44 (43.1%) and 47 patients (46.1%) on the tenth day after the treatment (*p* = 0.85); and 70 (68.6%) and 68 patients (67.6%) on the twentieth day after treatment (*p* = 0.46) in the groups treated with betadine and clotrimazole, respectively. The response to treatment was thus not significantly different in the two groups.

**Conclusion:**

In the present study the efficacy of betadine and clotrimazole was the same for the treatment of otomycosis. The result of this study supports the use of betadine as an effective antifungal in otomycosis treatment, helping to avoid the emergence of resistant organisms.

## Resumo

**Introdução:**

A otomicose é uma das doenças comuns associadas a muitas complicações, como envolvimento da orelha interna e mortalidade em casos raros. O tratamento da otomicose pode ser realmente desafiador e requer um acompanhamento rigoroso. As opções de tratamento para otomicose podem incluir desbridamento local, agentes antifúngicos locais e sistêmicos e uso de antissépticos tópicos, os medicamentos tópicos recomendados para o tratamento da otomicose.

**Objetivo:**

Comparar a taxa de recuperação de otomicose utilizando dois métodos terapêuticos de betadina tópica (Povidona-iodo) e clotrimazol.

**Método:**

Neste ensaio clínico simples cego, 204 pacientes com otomicose foram selecionados utilizando-se um método de amostragem de não probabilidade conveniente e randomizados para dois grupos de tratamento, com betadina tópica e com clotrimazol (102 pacientes em cada grupo). A resposta ao tratamento foi avaliada aos 4, 10 e 20 dias após o tratamento. Os dados foram analisados utilizando o teste t independente, qui-quadrado e teste exato de Fisher no software SPSS v.18, com nível de significância de *p* < 0,05.

**Resultados:**

Os resultados mostraram que dos 204 pacientes com otomicose, os tipos de fungos isolados incluíram *Aspergillus* em 151 casos (74%) e *Candida albicans* em 53 pacientes (26%). No quarto dia após o tratamento, 13 pacientes (13,1%) no grupo tratado com betadina e 10 pacientes (9,8%) no grupo tratado com clotrimazol apresentaram boa resposta ao tratamento (*p* = 0,75). Uma boa resposta ao tratamento foi relatada para 44 (43,1%) e 47 pacientes (46,1%) no décimo dia após o tratamento (*p* = 0,85); e 70 (68,6%) e 68 pacientes (67,6%) no vigésimo dia após o tratamento (*p* = 0,46) no grupo tratado com betadina e clotrimazol, respectivamente. Assim, a resposta ao tratamento não foi significativamente diferente nos dois grupos.

**Conclusão:**

No presente estudo, a eficácia da betadina e do clotrimazol foi a mesma no tratamento da otomicose. O resultado deste estudo apoia o uso de betadina como um antifúngico eficaz no tratamento da otomicose que pode ajudar a evitar o surgimento de organismos resistentes.

## Introduction

External ear infection is a common inflammation of the external ear canal and auricle that occurs due to various local infections factors or as a manifestation of a systemic disease. About 10% of people suffer from infection of the external ear canal in their lives, 90% of which is unilateral.^1^, ^2^ Fungal infection of external ear canal (Otomycosis) is a common disease accounting for 9%^3^, ^4^ to 27.2%^5^ of patients with signs and symptoms of external otitis media referred to Ear, Nose and Throat (ENT) centers. The infection is also the cause of more than 30% of patients with ear discharge and is a common cause of resistance to treatment of external otitis.^6^ Otomycosis is usually caused by predisposing factors, such as entry of foreign bodies into the ear canal, traumatic insemination of wood particles, plant materials and dirt into the ear canal, scratching and manipulation of the ear canal with non-sterile equipment, living in dusty areas or wet atmosphere, humidity of the ear canal after swimming and bathing, and fungal nail infection and dermatophitic lesions around the ear.^7^, ^8^

Otomycosis is associated with many complications including involvement of the inner ear with mortality in rare cases. Formation of a fungus ball or fungal mass of mycelia, epithelial cells, and wax in the ear canal: exposure of this mass to the tympanic membrane are causes of hearing loss. Neck skin and ear cartilage are also affected in acute infections. In chronic cases, a false veil of yeast in different colors (depending on the type of fungus) may occur in the ear canal. The mycosis can have a poor prognosis in immunocompromised individuals, especially in cases of cellular immunodeficiency and neutropenia. The disease presents many challenges, both for patients and for ENT specialists, and may recur despite long-term treatment and follow-up.^9^, ^10^, ^11^

The main treatment of otomycosis is the removal of visible debris and fungal elements. Topical medications recommended for the control of this condition include steroids, antiseptics, acidic solutions, antifungal agents and driers. Antifungal medications of otomycosis do not always cure the disease and in addition treatment should improve the physiological signs of external ear canal.^12^, ^13^ Using boric acid in an alcohol solution for the treatment of disease is associated with 23% recurrence rate. Furthermore, using antifungal solutions, such as clotrimazole or nystatin, may be effective for the treatment of *Candida* infections, but *Aspergillus* infections respond poorly to treatment.^14^, ^15^ This is while a wide range of fungi have been reported to cause otomycosis and the most common species is *Aspergillus*. Therefore, an appropriate treatment regimen is necessary for treatment. On the other hand, widespread and unnecessary use of antibacterial treatment for medial and external otitis may cause fungal overgrowth in this area, so the adverse effects of using wide-spectrum antibiotics are the secondary overgrowth of fungus and increasing prevalence of otomycosis.^16^, ^17^

Given the importance of otomycosis treatment as one of the challenges facing ENT specialists, this study aimed to compare the recovery rate of otomycosis using topical betadine and clotrimazole.

## Methods

### Study group

This single-blind clinical trial was conducted on 204 patients with a definitive diagnosis of otomycosis. After receiving permission from the Ethics Committee of the University and recording in Iranian Registry of Clinical Trials by the registration number (IRCT2014123020484N1), the participants were selected from those referred to the ENT clinic of Imam Reza and Vali-Asr Hospitals of Birjand University of Medical Sciences during the first six months of 2014. Patients with clinical signs and symptoms including pain, itching, mass in the external auditory canal, stuffy feeling associated with hearing loss and discharge were considered suspicious for otomycosis. The objectives of the study were explained to the participants and they were recruited into the study if they were willing to participate and had none of the exclusion criteria, including: otitis media with restriction of external ear canal, chronic mucus from ear, history of ear surgery, history of treatment with antifungal agents and corticosteroids, and external ear anomaly.

After recording the demographic characteristics such as age, sex, and obtaining informed consent, a total of 204 patients with otomycosis were enrolled into the study and recruited into one of the two treatment groups (102 patients in each group) by blocking randomization. All 204 enrolled patients signed informed consent forms.

### Collection of samples

Samples were taken by a special speculum from the mass in the ear canal. Furthermore, in the presence of pus and mucus, these were also separated in a sterile container and sent to specialized laboratories of mycology of Birjand University of Medical Sciences (BUMS).

### Mycological investigation

In the laboratory, the samples were evaluated by a microbiology specialist using KOH method, and the presence of fungal elements was considered as definite diagnosis of otomycosis.

A portion of the sample was spread on a clean slide glass for direct examination and another sample inoculated in the Sabouraud dextrose agar (Merck, Germany) supplemented with chloramphenicol (Fina Daru, Iran) medium for fungal growth. The plates were incubated at room temperature for two weeks. Fungi were identified by standard procedures.^18^ Furthermore, germ tubes on human sera and production of vesicles on corn meal agar (Merck, Germany) supplemented with tween 80 (Sigma-Aldrich, Germany) were done for the identification of yeast.

### Recovery rate of otomycosis

In the study, one patient group was treated with povidone-iodine so that at each visit, the patient's ear was washed by the physician using 10 mL betadine solution 10% with a syringe. The other group received 8 drops of antifungal clotrimazole, every eight hours. Patients were examined on 4, 10 and 20 days after treatment by an ENT specialist who did not know about the type of treatment. The patients were categorized into three groups based on clinical response: good response (dry external ear canal and the tympanic membrane and lack of secretion), partial response (slight discharge but not dry), and no answer (hypersecretion in the external auditory canal). If complete response, the treatment was discontinued; otherwise treatment was continued. Finally non-responders were considered treatment-resistant on the twentieth day and treatment regimen was continued with Tolnaftate and Violet de gentian.

### Statistical analysis

In the present study, to analyze the descriptive data, tables and charts of distribution frequency and for the inferential part, the independent *t*-test, Chi-square and Fisher exact test were used in SPSS v.18 software. The significant level was considered at *p* < 0.05.

## Results

In this study, a total of 204 patients with otomycosis were investigated in two treatment groups of betadine and clotrimazole (102 patients in each group). Overall, 86 (42.15%) of the patients with otomycosis were males and 118 (57.85%) were females. The average age of patients in two groups treated with betadine and clotrimazole was 38.61 ± 15.45 and 40.37 ± 16.15 years old, respectively. In this study, *Aspergillus* spp. was responsible for 75.4% and *Candida albicans* for 24.5% of otomycosis in the patient group treated with betadine. Prevalence of *Aspergillus* spp. and *C. albicans* was reported for 72.5 and 27.5 percentages of patients with otomycosis treated with clotrimazole.

Overall, the study showed no significant difference according to age (*p* = 0.43), sex (*p* = 0.4) and the causative agent of otomycosis (*p* = 0.63) between two treatment groups of betadine and clotrimazole (Table 1).

**Table 1:** Comparison of demographic characteristics and distribution of fungi in patients with otomycosis based on the treatment type.

Demographic characteristics	Topical betadine group (%)	Topical clotrimazole group (%)	*p*-Value
*Age (years)*^a^	38.61 ± 15.45	40.37 ± 16.15	0.43
*Gender*			0.40
Male	40 (39.2)	46 (45.1)	
Female	62 (60.8)	56 (54.9)	

*Fungal agent*			0.63
*Aspergillus* spp.	77 (75.4)	74 (72.5)	
*Candida albicans*	25 (24.5)	28 (27.5)	

a Values in table are mean ± SD.

In the study, recovery rate of otomycosis was evaluated in two treatment groups of betadine and clotrimazole. The results demonstrated that on the fourth day after treatment, 13 patients (13.1%) in the group treated with betadine and 10 patients (9.8%) in the group treated with clotrimazole had a good response to treatment (*p* = 0.75). A good response to treatment was reported for 44 (43.1%) and 47 patients (46.1%) on the tenth day after the treatment (*p* = 0.85); and 70 (68.6%) and 68 patients (67.6%) on the twentieth day after treatment (*p* = 0.46) in the group treated with betadine and clotrimazole, respectively. It is noteworthy that in none of the patients treated with betadine any side effects were observed.

In our study, there was no statistically significant difference in terms of response to treatment in the fourth, tenth and twentieth day after treatment between two treatment groups of betadine and clotrimazole (*p* < 0.05) (Table 2).

**Table 2:** Comparison of response to treatment on the fourth, tenth and twentieth day after treatment in patients based on the treatment type.

Course of treatment	Type of treatment	Response to treatment			*p*-Value
		No response (%)	Partial response (%)	Good response (%)	
4 days	Betadine^a^	31 (31.3)	55 (55.6)	13 (13.1)	0.75
	Clotrimazole	32 (31.4)	60 (58.8)	10 (9.8)	

10 days	Betadine	8 (7.8)	50 (49)	44 (43.1)	0.85
	Clotrimazole	9 (8.8)	46 (45.1)	47 (46.1)	

20 days	Betadine	13 (12.7)	19 (18.6)	70 (68.6)	0.46
	Clotrimazole	9 (8.8)	25 (24.5)	68 (66.7)	

a Three patients did not attend on the fourth day after the treatment.

## Discussion

Otomycosis is one of the common diseases that are associated with many complications including involvement of the inner ear and mortality in rare cases. The disease presents many challenges, both for patients and for ENT specialists, and may recur despite the concern for long-term treatment and follow-up.^9^, ^11^

Results of this study showed that among 204 patients with otomycosis, *Aspergillus* spp. was responsible for 74% (151 cases) and *C. albicans* for 26% (53 patients) of the infection. In the Pradhan et al. study, *Aspergillus* species and *C. albicans* were reported as the most common species of fungi isolated from patients with otomycosis in 2003.^19^ The study conducted by Satish and colleagues showed that *Aspergillus* species (77%) was the most commonly isolated fungus in the immunocompetent group while *Candida* (53.4%) was commonly isolated in the immunocompromised group.^20^ In another study on 95 patients suspected of having the fungal infection, 72 cases of fungal cultures were positive and *Aspergillus* was identified as the most common fungus grown in culture (41.1%) and *C. albicans* was in the next grade, with a prevalence of 8.2%.^21^ The results of our study were consistent with the results above. *Aspergillus* species are one of the most common causes of opportunistic invasive fungal infections, especially otomycosis. This may be due to its high prevalence in dust and the acidic nature of the ear canal, as *Aspergillus* species grow in pH 5 to 7.^22^

After completion of the treatment course in the present study, the results showed that on the fourth day after treatment, 55.6% and 58.8% of patients treated with betadine and clotrimazole had partial response to treatment, respectively. In addition, on the tenth day after treatment, 49% of patients treated with povidone-iodine had partial response to treatment, and 46.1% of patients had a good response to treatment with clotrimazole. Finally, on the twentieth day after treatment, 68.6% of patients treated with betadine and 66.7% of patients treated with clotrimazole had good response to treatment. Overall, in our study there was no statistically significant difference in terms of response to treatment in the fourth, tenth and twentieth day after treatment between two treatment groups of betadine and clotrimazole. Regarding the antifungal treatment effect on otomycosis, several studies have been conducted so far. In a single-blind randomized clinical study, the effect of betadine 7.5% in comparison with clotrimazole 1% and lignocaine was evaluated on patients with otomycosis. After the treatment period, in the betadine patients group, the symptoms of itching, discharge from the ear, ear fullness, tinnitus and deafness were treated in 83.3%, 97.1%, 83.3%, 91.7%, and 91.7% of cases, and ear pain was treated in 100% of cases. While, in patients treated with clotrimazole and lignocaine drops, the symptoms of itching, ear discharge and fullness were cured in 93.3%, ear pain in 86.7% and tinnitus in 100% of cases.^23^ In another study, the therapeutic effect of miconazole ointment and clotrimazole drop was compared in patients with otomycosis. All patients were examined for response to treatment one and two weeks after treatment. The results of the final analysis showed that and there was no difference in terms of response to treatment between the two treatment groups.^24^ Stern and colleagues demonstrated that clotrimazole was effective against most yeasts and molds but tolnaftate had no impact on otomycosis.^25^ In another study, clotrimazole and econazol were cited as the drug of choice in the treatment of otomycosis.^26^

According to the above-mentioned issues, it can be declared that the treatment of otomycosis is today one of the challenges facing ENT specialists, as a great number of fungal infections of external ear are resistant to the available antifungal drugs. Otomycosis treatment requires early detection and timely treatment of patients regarding the possibility of drug resistance in chronic infections. It should be noted that the basis of the treatment of otomycosis is keeping the ear dry and the ear hygiene, and the appropriate treatment protocol should be considered according to the different types of fungi causing the disease and sensitivity to different antifungal drugs.^16^, ^27^ In our study, clotrimazole and povidone-iodine were used as two drug regimens for treatment of otomycosis. Clotrimazole is a medicine containing azole groups that is used to treat infections. Betadine is also a remedy for infections that is easily accessible and its effect has been proven on chronic suppurative otitis media as the precipitating factors of otomycosis. This drug is a stable, inexpensive substance, and bacterial and fungal resistance to it has not yet been reported. Therefore, betadine can be a good choice for otomycosis treatment in developing countries, due to its low cost, effectiveness and lack of ototoxicity.^28^, ^29^

## Conclusion

According to the results, efficacy of betadine and clotrimazole regimens was identical in treatment of otomycosis. Our findings reinforce the use of betadine for otomycosis treatment due to cost-effectiveness and appropriate therapeutic effect on *Aspergillus* species and *C. albicans*, the most common causes of otomycosis. Such treatment can help to avoid the emergence of resistant organisms.

## Conflicts of interest

The authors declare no conflicts of interest.
